# High-resolution mapping reveals a *Ht3*-like locus against northern corn leaf blight

**DOI:** 10.3389/fpls.2022.968924

**Published:** 2022-09-09

**Authors:** Mang Zhu, Jun Ma, Xinfang Liu, Yanling Guo, Xin Qi, Xue Gong, Yanbin Zhu, Yanbo Wang, Min Jiang

**Affiliations:** ^1^Liaoning Academy of Agricultural Sciences, Shenyang, China; ^2^State Key Laboratory of Plant Physiology and Biochemistry, College of Agronomy and Biotechnology, National Maize Improvement Center, Center for Crop Functional Genomics and Molecular Breeding, China Agricultural University, Beijing, China; ^3^Liaoning Dongya Agricultural Development Co., Ltd., Shenyang, China

**Keywords:** *Ht3*, northern corn leaf blight (NCLB), maize (*Zea mays* L.), maize disease, fine-mapping

## Abstract

Northern corn leaf blight (NCLB), caused by the fungal pathogen *Exserohilum turcicum*, poses a grave threat to maize production worldwide. The resistance gene in A619*Ht3*, discovered decades ago, is an important genetic resource for NCLB control. By using a pair of near-isogenic lines (NILs) A619*Ht3* and A619, together with the resistant and susceptible bulks derived from the cross of A619*Ht3* and L3162 lines, we initially detected a *Ht3*-like (*Ht3L*) locus in bin 8.06 that was closely associated with NCLB resistance. We then performed five rounds of fine-mapping, which ultimately delimited the *Ht3L* locus to a 577-kb interval flanked by SNP markers KA002081 and KA002084. Plants homozygous for the *Ht3L*/*Ht3L* genotype exhibited an average reduction in diseased leaf area (DLA) by 16.5% compared to plants lacking *Ht3L locus*. The *Ht3L* locus showed extensive variation in genomic architecture among different maize lines and did not appear to contain any genes encoding canonical cell wall-associated kinases against NCLB. Moreover, the *Ht3L* locus was located ∼2.7 Mb away from the known *Htn1* locus. We speculate that the *Ht3L* locus may contain a bona fide *Ht3* gene or a novel NCLB resistance gene closely linked to *Ht3*. In practice, the *Ht3L* locus is a valuable resource for improving maize resistance to NCLB.

## Introduction

Northern corn leaf blight (NCLB), caused by the hemibiotrophic fungus *Exserohilum turcicum* (Chang and Fan, 1986), is one of the most devastating foliar diseases in most maize-growing areas worldwide. Maize (*Zea mays* L.) grown in areas with high humidity and moderate temperatures is more prone to NCLB outbreaks. During the grain-filling period, NCLB causes leaf necrosis and thus abolishes photosynthetic output, leading to lower grain yield (Raymundo and Hooker, 1981). The development and deployment of resistant maize varieties is the most environmentally friendly and cost-effective way to reduce yield loss caused by NCLB.

Maize resistance to NCLB is a very complex trait, including both qualitative and quantitative resistance. Several qualitative resistance genes against NCLB have been discovered. The first locus, *Helminthosporium turcium resistance l* (*Ht1*), was identified in the inbred line ‘GE440’ and ‘Ladyfinger’ popcorn in 1959; this locus shows a partially dominant inheritance pattern and maps to chromosome 2L (Bentolila et al., 1991). *Ht1* substantially inhibits the formation of conidia in chlorosis (Hilu and Hooker, 1964, 1965). The *Ht2* locus displays a similar resistance performance and genetic architecture to *Ht1* and maps to chromosome 8L (Hooker, 1977). *Ht3*, a dominant single gene identified independently of *Ht1* and *Ht2*, was introgressed into the maize genome from the wild maize relative Florida gamagrass (*Tripsacum floridanum*) (Hooker, 1981). *Ht2* and *Ht3* were recently shown to be identical and allelic to the previously cloned *Htn1* gene (Yang et al., 2021). At the maturity stage, plants harboring *Ht2* or *Ht3* display necrotic and chlorotic lesions, respectively, while plants with *Htn1* show a delay in lesion formation (Hooker, 1977, 1981; Welz and Geiger, 2000). This observation suggests that other genes may be linked to *Htn1* and contribute to the response against NCLB. Moreover, histological studies revealed that plants with different resistance genes (*Ht1*, *Ht2*, *Ht3*, or *Htn1*) show different symptoms, indicating that resistance mechanisms conferred by these genes are not equivalent (Navarro et al., 2020).

Previous studies have shown that quantitative trait loci (QTLs) for resistance to NCLB are dispersed over all 10 chromosomes in maize (Welz and Geiger, 2000; Wisser et al., 2006; Balint-Kurti et al., 2010; Zwonitzer et al., 2010). Although many QTLs associated with resistance against NCLB have been identified in different populations, few have been fine-mapped or their causal genes even cloned. For instance, a major QTL was detected on chromosome 8 from a cross derived from two near-isogenic lines (NILs) with contrasting performance for NCLB resistance and fine-mapped to a 460-kb region containing 12 annotated genes (Chung et al., 2010). Another major QTL, designated *qNLB1.06*, was anchored to a 3.6-Mb region and narrowed down to two putative candidate genes by joint linkage and association mapping (Jamann et al., 2014). The QTL *qNCLB7.02* was mapped to chromosome 7 by linkage mapping and validated in chromosome segment substitution lines (CSSLs) (Wang et al., 2018). Single-marker and haplotype-based association mapping studies identified 12 and 10 loci, respectively, that were significantly associated with NCLB resistance (Ding et al., 2015). A total of 29 resistance QTLs against NCLB were identified using a nested association mapping (NAM) population with 5,000 recombinant inbred lines (RILs) (Poland et al., 2011). To date, the gene *ZmWAK-RLK1* (*Wall-associated-receptor-like kinase 1*) at the *Htn1* locus is the only resistance QTL against NCLB that has been cloned through map-based cloning (Hurni et al., 2015). Further investigation revealed that NCLB resistance mediated by ZmWAK-RLK1 correlates with reduced benzoxazinoid contents (Yang et al., 2019).

Although the *Htn1* locus is effective against most prevalent NCLB races, NCLB isolates with virulence in *Htn1*-bearing plants have been reported (Weems and Bradley, 2018; Jindal et al., 2019). Thus, there is an urgent need to explore novel loci conferring NCLB resistance. Quantitative disease resistance has been widely utilized in resistance breeding programs due to its moderate effectiveness and the durable and non-race-specific resistance it confers (Ayliffe et al., 2008; Poland et al., 2009). Introgression of both qualitative and quantitative resistance loci into inbred lines via marker-assisted backcrossing (MABC) is a powerful means to control diseases in maize (Zhao et al., 2012; Li et al., 2017).

Bulked-segregant analysis (BSA) is a rapid, technically simple method to identify markers linked to specific genes (Michelmore et al., 1991), which has been widely used in rice (*Oryza sativa*) (Venuprasad et al., 2009), wheat (*Triticum aestivum*) (Shen et al., 2003) and maize (Cai et al., 2003; Li et al., 2018). Another powerful strategy to narrow down a QTL interval is sequential QTL fine-mapping based on the genotypes and phenotypes of progeny derived from recombinants (Yang et al., 2012). Many quantitative disease-resistance genes have been cloned using this strategy (Zuo et al., 2015; Leng et al., 2017; Liu et al., 2017, 2020; Wang et al., 2017; Ye et al., 2019). In the current study, we conducted QTL identification and high-resolution mapping of the *Ht3*-like (*Ht3L*) locus in the resistant line A619*Ht3* by combining NIL analysis, BSA and sequential QTL fine-mapping. Our results lay the foundation for the future map-based cloning of the causal gene at the *Ht3L* locus. The molecular markers on or adjacent to *Ht3L* described here can also be used for MABC to improve maize resistance to NCLB in breeding programs.

## Materials and methods

### Plant materials

The pair of near-isogenic lines (NILs) A619*Ht3* and A619 was obtained from the Eastern Cereal and Oilseed Research Centre of Agriculture and Agri-Food Canada. While A619*Ht3* is highly resistant to NCLB, A619 is highly susceptible to NCLB, and both NILs share ∼98.84% genomic homozygosity (Ma et al., 2014). The elite but susceptible inbred line L3162 is the male parent of the hybrids Liaodan565 and Liaodan566 widely grown in China. From a cross between A619*Ht3* (donor parent) and L3162 (recurrent parent), multiple backcross populations were developed for initial QTL detection and fine-mapping. In 2016, the two parental lines (A619*Ht3* and L3162) and their BC_2_F_1_ backcross population comprising 362 individuals were grown at the Shenyang experimental station (41°46′N, 123°26′E) in China for artificial inoculation. Twenty-nine highly susceptible and 29 highly resistant plants were selected to form the susceptible and resistant bulks, respectively, for initial mapping of the *Ht3L* locus. Subsequently, the BC_3_F_1_ progeny from recombination events in the mapped *Ht3L* region were identified and backcrossed to L3162 (Hainan winter nursery, 18°39′N and 109°21′E) to produce BC_4_F_1_ progeny for fine-mapping. In the following five rounds of fine-mapping from 2017 to 2021, newly-occurring recombinants were screened in Shenyang and then backcrossed twice to L3162 in Shenyang and Hainan to generate progeny. All offspring were investigated for NCLB resistance at the Shenyang experimental station. A619*Ht3* and L3162 were planted annually in each experimental block as resistant and susceptible controls, respectively.

### Artificial inoculation and northern corn leaf blight disease evaluation

*Exserohilum turcicum* (mixed races 0, 1, 2, 3N, 12N, and 23N) was cultured on potato dextrose agar (PDA) medium for 15 days at 25°C and then stored in the dark at room temperature. Plant materials were artificially inoculated at the V8-V10 leaf stages with ∼5 mL of spore suspension (1 × 10^5^-10^6^ conidia per mL in 0.02% [v/v] Tween 20) per plant. Spray irrigation was then performed for 2 days following inoculation, three to four times a day, to maintain a humid environment. Four weeks after inoculation, NCLB lesions were prominent and suitable for first scoring, followed by two more scorings over the next 14 days. Diseased leaf area (DLA) was examined for three leaves (ear leaf and its upper and lower leaves) based on standards listed in Supplementary Figure 1. Average DLA was used to represent NCLB severity.

### Genotyping

Fresh leaf tissue at the five-leaf seedling stage was harvested for high-throughput extraction of genomic DNA in 96-well plates. Plant genomic DNA was extracted according to the method described previously (Murray and Thompson, 1980). The Maize3K Chip was used to genotype the NILs (A619*Ht3* and A619) and the two parental lines (A619*Ht3* and L3162). The Maize6K Chip was used to genotype each plant in the selected susceptible and resistant bulks. Kompetitive Allele-Specific PCR (KASP) markers, developed in the target *Ht3L* region, were subjected to automated genotyping with the QuantStudio 12 K Flex Real-Time PCR System (Applied Biosystems by Life Technologies).

### Bulk-based association analysis and statistical analysis

The single-nucleotide polymorphism (SNP) index was calculated based on the heterozygosity at each SNP within a given bulk. For each SNP, the Δ(SNP-index) value was calculated using the following formula: Δ(SNP-index) = SNP-index in the resistant bulk – SNP-index in the susceptible bulk (Takagi et al., 2013). The Δ(SNP-index) value was used to identify candidate regions for *Ht3L*. The average Δ(SNP-index) value of the SNPs located in each genomic interval was calculated using a sliding window with a window size of 10 Mb and 10-kb steps. The distribution of Δ(SNP-index) along the chromosomes was plotted using R software. SNP markers in the candidate *Ht3L* regions were also used to conduct regional association analyses. For each SNP marker, the significant difference in SNP-index between two bulks was determined by chi-squared test.

### Genomic structure analysis

Markers in the *Ht3L* region were identified according to the B73 reference genome sequence and were projected onto the 25 founders of the NAM population to define their corresponding *Ht3L* regions. The sequence information and annotated genes in the *Ht3L* regions were then retrieved from MaizeGDB^1^. The gene distribution in susceptible and resistant founders was plotted using R software.

## Results

### A619*Ht3* exhibits a distinct resistance spectrum to *Exserohilum turcicum* races

To determine the A619*Ht3* resistance spectrum, we conducted virulence testing on the five near-isogenic lines containing the different *Ht* loci (A619, A619*Ht1*, A619*Ht2*, A619*Ht3* and A619*HtN*) by inoculating designated physiological races of *Exserohilum turcicum* (1,2,3, and 123N) in growth chamber. The results showed that A619*Ht3* exhibits susceptible to races 3 and 123N, while resistant to races 1 and 2 (Table 1), which was consistent with the resistance spectrum of the *Ht3* locus in OH43 and HZS genetic backgrounds (Guo, 2015). We thus named the resistance gene in A619*Ht3* as *Ht3*-like (*Ht3L*) to distinguish it from the recently reported *Ht3* allele that is identical to *Ht2* and allelic to *Htn1* (Yang et al., 2021).

**TABLE 1:** Virulence testing on the five different *Ht* genes by designating physiological races of *Exserohilum turcium*.

Race	Response				
	A619	A619*Ht1*	A619*Ht2*	A619*Ht3*	A619*HtN*
1	S	S	R	R	R
2	S	R	S	R	R
3	S	R	R	S	R
123N	S	S	S	S	S

R: resistant, no necrosis, or chlorotic lesions surrounded by chlorosis; S: susceptible, necrotic lesions without chlorotic border.

### Preliminary mapping of the *Ht3L* locus

**FIGURE 1:**
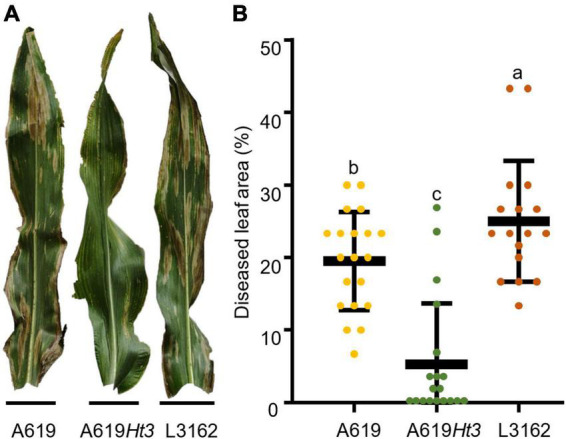
Performances of three maize inbred lines A619, A619*Ht3*, and L3162 against NCLB at nearly 6 weeks after inoculation in 2021. A619*Ht3* exhibited highly resistant to NCLB, while A619 and L3162 show highly susceptible to NCLB. **(A)** Symptoms of NCLB on leaves; **(B)** Diseased leaf area (DLA).

The NILs A619 and A619*Ht3* differ considerably in their resistance to NCLB, with A619 being highly susceptible and A619*Ht3* extremely resistant (Figures 1A,B). We genotyped the A619 and A619*Ht3* NILs using the Maize3K Chip to identify chromosomal region(s) potentially covering the *Ht3L* locus. We detected three segments with stretches of polymorphisms between the two NILs in bins 3.08 (6 SNPs), 7.03/7.04 (12 SNPs) and 8.06 (12 SNPs) (Table 2), which we considered candidate *Ht3L* regions.

**TABLE 2:** Three candidate *Ht3*-like regions associated with NCLB resistance.

Chr.	Flanking markers		Bins	Physical position (bp, AGPv5)		Number of SNPs	Magnitude (bp)
				Start point	End point		
3	PZE-103160158	SYN8639	3.08	216,799,618	222,134,027	6	5,334,409
7	PUT-163a-76010550-3720	PZE-107107154	7.03/7.04	154,680,564	167,990,718	12	13,310,154
8	PZE-108096541	PZE-108110136	8.06	156,678,533	169,300,416	12	12,621,883

To determine which segment harbors the *Ht3L* locus, we selected the susceptible line L3162 that is genetically distinct from A619*Ht3* to generate mapping populations. We genotyped A619*Ht3* and L3162 with the Maize3K Chip, which returned 493 SNPs distributed over all maize chromosomes, numbers sufficient for an initial mapping of *Ht3L* (Supplementary Table 1). Within the frames of three *Ht3L* candidate regions defined by two NILs, we searched for SNPs between two parental lines A619*Ht3* and L3162 to define the cognate *Ht3L* candidate regions.

**FIGURE 2:**
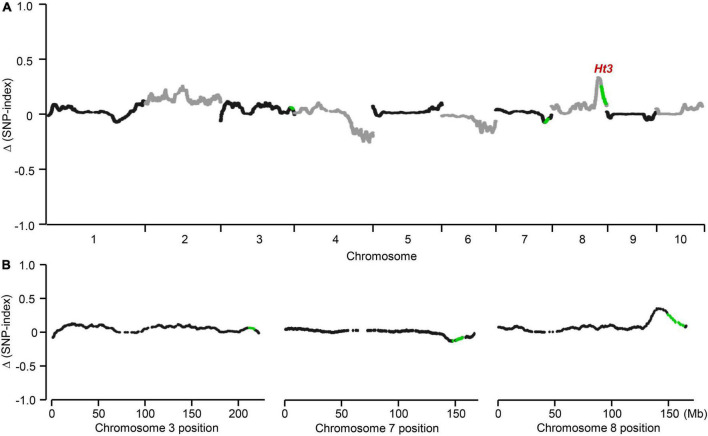
Initial detection of the *Ht3L* locus. In the BC_2_F_1_ segregating population, highly resistant and susceptible individuals were selected to prepare two blocks. SNP-index value which represents the heterozygosity was estimated for each SNP. **(A)** Genome-wide association mapping of *Ht3L* locus. **(B)** *Ht3L* was located in bin 8.06. Difference in SNP-index between two bulks, Δ(SNP-index), was calculated for all SNPs and used for genome-wide association. The average Δ(SNP-index) was calculated using a sliding window analysis with 10-Mb window size and 10-kb increments. Each plot represents a SNP marker, and the green plots correspond to the significantly associated SNPs in NILs analysis. The *Ht3L* locus was identified as a peak of the Δ(SNP-index) in bin 8.06.

From a BC_2_F_1_ population derived from a cross between A619*Ht3* and L3162, we selected 29 highly resistant and 29 susceptible individuals to prepare two bulks. We then used the Maize6K Chip to genotype each BC_2_F_1_ individual from the two bulks and calculated the Δ(SNP-index) for each SNP in the resistant and susceptible bulks. This analysis revealed a prominent peak on chromosome 8 (Figure 2A). We also calculated the SNP-index values for those SNPs mapping to the three *Ht3L* candidate regions defined above between the two bulks. We detected no significant difference for SNP-index values between the two bulks within bins 3.08 or 7.03/7.04 (Figure 2B and Supplementary Table 2). By contrast, the SNP-index value for every SNP in bin 8.06 was significantly higher in the resistant bulk than in the susceptible bulk, indicating that the *Ht3L* locus might lie within bin 8.06 (Figure 2B and Table 3). Taken together, we concluded that the *Ht3L* locus maps to bin 8.06, which was flanked by SNP markers PZE-108095959 and PZE-108110343 and represented a physical distance of 11.23 Mb based on the B73 physical map (AGPv5).

**TABLE 3:** SNP markers in bin 8.06 significantly associated with NCLB resistance.

Chr.	Marker	Bins	Physical position (bp, AGPv5)	SNP-index (%)		χ^2^	*P*-value
				Resistant bulk	Susceptible bulk		
8	PZE-108095959	8.06	156,276,743	0.62	0.28	5.6	0.0083
	PZE-108097802	8.06	157,706,869	0.62	0.28	5.6	0.0083
	PZE-108097921	8.06	157,881,029	0.62	0.28	5.6	0.0083
	PZE-108098977	8.06	159,229,224	0.59	0.28	4.5	0.0170
	PZE-108099332	8.06	159,625,544	0.62	0.28	5.6	0.0083
	PZE-108099526	8.06	159,865,570	0.62	0.28	5.6	0.0083
	PZE-108099959	8.06	160,066,656	0.62	0.28	5.6	0.0083
	PZE-108103023	8.06	164,969,666	0.59	0.31	3.4	0.0347
	PZE-108103951	8.06	165,599,868	0.59	0.31	3.4	0.0347
	PZE-108105699	8.06	166,329,198	0.59	0.31	3.4	0.0347
	SYN32657	8.06	166,526,926	0.59	0.31	3.4	0.0347
	PZE-108106737	8.06	166,828,537	0.59	0.31	3.4	0.0347
	SYN10384	8.06	168,773,685	0.59	0.31	3.4	0.0347
	SYN13369	8.06	169,143,087	0.59	0.31	3.4	0.0347
	PZE-108110343	8.06	169,514,058	0.59	0.31	3.4	0.0347

### Development of high-density molecular markers in the *Ht3L* region

We developed high-density molecular markers over the *Ht3L* region to saturate the target region. Accordingly, we retrieved the SNP markers in the *Ht3L* region between A619*Ht3* and L3162 from the genotype data of the Maize6K Chip. We then converted all SNPs located in the *Ht3L* region into KASP markers and tested them against A619*Ht3* and L3162. Totally, we obtained 18 effective KASP markers for fine-mapping (Supplementary Table 3).

### Sequential fine-mapping of the *Ht3L* locus

**FIGURE 3:**
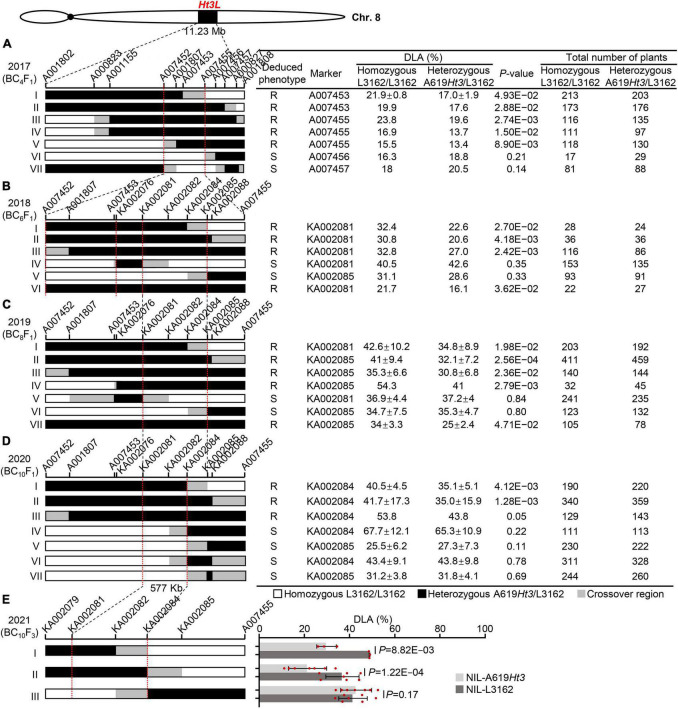
Sequential fine-mapping of the *Ht3L* locus by using the recombinant-derived progeny. The vertical bars mark the sites of key molecular markers. The red dotted lines indicate the left and right boundaries of the mapped *Ht3L*. The chromosomal composition at *Ht3L* is depicted as black, white, and gray rectangles, representing heterozygous A619*Ht3*/L3162, homozygous L3162/L3162, and recombination breakpoint regions, respectively. The total number of plants refers to all progeny of a given recombinant. The significant difference in DLA among genotypes was calculated using *t-*test. A significant difference in DLA (*P* < 0.05) between heterozygous and homozygous offsprings indicated the presence of *Ht3L* in the A619*Ht3* donor region, and the corresponding parental recombinants were deduced to be NCLB resistance (R). A *P*-value > 0.05 indicates that no significant difference in DLA between heterozygous and homozygous offsprings, suggesting the absence of *Ht3L* in the donor region, and the corresponding recombinants were deduced to be NCLB susceptibility (S). *Ht3L* was narrowed from an ∼11.23-Mb to an ∼577-kb region flanked by the markers KA002081 and KA002084 through five rounds of fine-mapping process. **(A)** *Ht3L* was initially mapped in bin 8.06 with the physical distance of 11.23-Mb and fine-mapped to an ∼2.17-Mb interval with seven BC_4_F_1_ recombinants. **(B)** *Ht3L* was localized into either A007452/KA002076 or KA002081/KA002085 intervals by using six BC_6_F_1_ recombinants. **(C)** *Ht3L* was confirmed to be located into the 838-kb KA002081/KA002085 interval by using seven BC_8_F_1_ recombinants. **(D)** *Ht3L* was narrowed down to a 577-kb interval flanked by markers KA002081 and KA002084 by using seven BC_10_F_1_ recombinants. **(E)** *Ht3L* was further confirmed to be in a 577-kb interval by using three pairs of NILs.

We used a sequential fine-mapping strategy based on recombinant-derived progeny to narrow down the location of the *Ht3L* locus. In the first round of fine-mapping, we used six KASP markers (A001802, A000823, A001155, A001807, A000827, and A001808) to detect recombination events in the 11.23-Mb *Ht3L* region from the BC_2_F_1_ population obtained in 2016. We identified seven BC_2_F_1_ recombinants, which we backcrossed twice to the susceptible parent L3162 to produce their corresponding BC_4_F_1_ populations. With an additional five newly-developed KASP markers (A007452, A007453, A007455, A007456, and A007457), we determined the precise recombination breakpoint for each recombinant. To this end, we grew 1,678 plants from the BC_4_F_1_ progeny in the field and scored them for their extent of diseased leaf area (DLA) in Shenyang in 2017 (Figure 3A). In parallel, the marker in the heterozygous *Ht3L* region was used to genotype all individuals to distinguish homozygous (L3162/L3162) from heterozygous (A619*Ht3*/L3162) BC_4_F_1_ plants. With the both genotypic and phenotypic data, an average DLA value can be estimated for both homozygous and heterozygous genotypes in each BC_4_F_1_ progeny. Significant difference in DLA between two genotypes indicated the presence of the *Ht3L* locus in the A619*Ht3* donor segment; otherwise, there is no *Ht3L* locus. As shown, recombinants I to V showed a significant difference (*P* < 0.05) in their DLA values between homozygous and heterozygous genotypes in their BC_4_F_1_ progeny, indicating that they carried the *Ht3L* locus in the A619*Ht3* donor segment (Figure 3A), while recombinants VI and VII exhibited no significant difference (*P* > 0.05), and thus lacked the *Ht3L* locus. Recombinants V (with *Ht3L*) and VII (without *Ht3L*) defined the left boundary of the mapping interval to marker A007452, while recombinants I (with *Ht3L*) and VI (without *Ht3L*) marked the right boundary with marker A007455. The resulting interval spanned a region of 2.17 Mb (AGPv5) (Figure 3A).

Based on the results shown in Figure 3A, we selected those recombinants with crossovers within the 2.17-Mb mapping interval for the next round of fine-mapping, as such recombinants were still valuable to resolve the *Ht3L* locus with high-density markers. Thus, we backcrossed recombinants I and V to L3162 twice to produce their BC_6_F_1_ progeny. We also identified another three new recombinants within the 2.17-Mb region from the BC_4_F_1_ progeny, which, together with a heterozygous plant (as a positive control), were backcrossed twice to L3162 to produce BC_6_F_1_ progeny. We genotyped the five resulting BC_6_F_1_ progeny consisting of 847 individuals, grown in the field for NCLB testing in 2018, with 11 markers spanning the 2.17-Mb region, including seven newly-developed markers. We also tested the markers against the heterozygous *Ht3L* region to distinguish homozygous from heterozygous BC_6_F_1_ progeny. We detected a significant difference (*P* < 0.05) in DLA for recombinants I, II and III, together with the positive control (VI in Figure 3B), between the two genotypes in their BC_6_F_1_ progeny, but not for recombinants IV or V. Thus, the recombinant III defines a new left boundary of the mapping interval with marker A007452, and the recombinants I and V define a new right boundary with marker KA002085. Notably, recombinant IV appeared to harbor two crossovers that allowed us to exclude the region between markers KA002076 and KA002081 for the *Ht3L* locus, thus delineating the *Ht3L* locus to either the A007452-KA002076 or KA002081-KA002085 interval (Figure 3B).

From the BC_6_F_1_ populations, we isolated one new recombinant that, along with six existing BC_6_F_1_recombinants, was backcrossed twice to L3162 to produce seven BC_8_F_1_ populations comprising 2,540 individuals. With this third round of fine-mapping, we observed that the new recombinant (IV in Figure 3C) shows a significant difference in DLA between the homozygous and heterozygous BC_8_F_1_ offspring. This recombinant carried a heterozygous region downstream of marker KA002076, which excluded the A007452-KA002076 interval as the candidate region (Figure 3C). The other six BC_8_F_1_ progeny derived from previous recombinants exhibited similar results, with recombinants I to III harboring the *Ht3L* locus in their heterozygous region, but not recombinants V or VI. Taken together, this third round of fine-mapping delimited the *Ht3L* locus to the region flanked by markers KA002081 and KA002085, with a physical length of 838 kb (AGPv5).

In the summer of 2020, we planted 3,200 BC_10_F_1_ plants corresponding to seven recombinants in Shenyang for further fine-mapping. Of them, two new recombinants (IV and VI in the Figure 3D) with breakpoints between KA002082 and KA002084 showed no significant difference (*P* > 0.05) in DLA between the BC_10_F_1_ homozygous and heterozygous genotypes, indicating the A619*Ht3* donor region lacked *Ht3L*. Based on these two recombinants, we moved the right boundary from marker KA002085 to marker KA002084. Phenotypic scorings for NCLB severity in the field indicated that the progeny from recombinants I-III showed a significant difference (*P* < 0.05) in DLA between the homozygotes and heterozygotes in their BC_10_F_1_ progeny, whereas the progeny from recombinants IV–VII did not show this difference. Thus, we ultimately anchored *Ht3L* to a 577-kb interval flanked by markers KA002081 and KA002084 (AGPv5).

From the BC_10_F_1_ progeny, we selfed heterozygous recombinants to develop three pairs of NILs, which were planted in the summer of 2021 to evaluate their DLA values. The first two pairs of NILs (I and II in Figure 3E) displayed significant differences in the DLA scores between NILs with and without A619*Ht3* donors, but the third pair did not (Figure 3E). These results confirmed that *Ht3L* maps to the 577-kb interval flanked by markers KA002081 and KA002084 (AGPv5).

### The genetic effect of *Ht3L* locus in resistance to northern corn leaf blight

**FIGURE 4:**
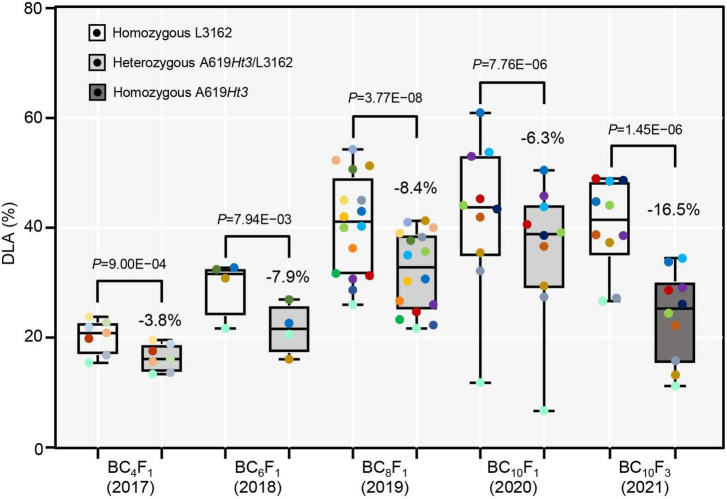
The genetic effect of the *Ht3L* locus. The DLAs are shown for both homozygous and heterozygous genotypes at *Ht3L* in the BC_4_F_1_, BC_6_F_1_, BC_8_F_1_, BC_10_F_1_ populations. The difference in DLA homozygous *ht3l/ht3l* and heterozygous *Ht3L/ht3l* genotypes was calculated for each generation. In the last two columns, the DLAs were calculated for two homozygous genotypes *ht3l/ht3l* and *Ht3L/Ht3L* of NIL-I and NIL-II. The *P* values between two genotypes were calculated by paired two-tailed *t*-test and indicated. *P*-value < 0.05 indicates significant difference; *P*-value > 0.05 indicates no significant difference.

We calculated the genetic effect of the *Ht3L/ht3l* genotype as the difference in the DLA values between heterozygous and homozygous genotypes for each of the BC_4_F_1_, BC_6_F_1_, BC_8_F_1_ and BC_10_F_1_ progeny. For the *ht3l/ht3l* and *Ht3L/ht3l* genotypes, we estimated the average DLA values to be 20.3% and 16.5% in BC_4_F_1_, 29.4% and 21.6% in BC_6_F_1_, 40.5% and 32.1% in BC_8_F_1_ and 42.2% and 35.9% in BC_10_F_1_, respectively (Figure 4). As expected, the *Ht3L/ht3l* heterozygotes had lower DLA values than *ht3l/ht3l* homozygotes. We also estimated the genetic effect of *Ht3L/ht3l* relative to *ht3l/ht3l*: 3.8% in BC_4_F_1_, 7.9% in BC_6_F_1_, 8.4% in BC_8_F_1_ and 6.3% in BC_10_F_1_ progeny, respectively. For the three pairs of NILs, the average DLA values of the susceptible NILs (with *ht3l/ht3l*) and resistant NILs (with *Ht3L/Ht3L*) were 40.4% and 23.9%, respectively. Thus, the genetic effect of the homozygous *Ht3L/Ht3L* genotype was 16.5% (Figure 4).

### Exploration of the annotated genes in the mapped *Ht3L* region across various maize lines

**FIGURE 5:**
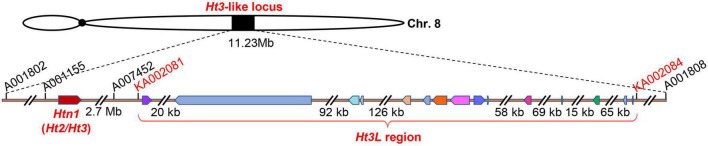
The *Ht3L* locus is independent from *Htn1*. The mapped *Ht3L* region encompassing 15 annotated genes, which was located 2.7-Mb to the right of *Htn1*.

Bin 8.06 is associated with several *Ht* genes, such as *Ht2* and *Htn1*. The identified resistance genes belong to the cell wall-associated-like kinase gene family and exhibit extensive variation in their genomic structure (Yang et al., 2021). Within the current 577-kb mapping interval of *Ht3L* locus, we detected 15 annotated genes according to the B73 reference genome sequence (RefGen_v5, Figure 5 and Table 4). To our surprise, we identified no *WAK-like* gene within this interval, which appeared inconsistent with a recent report (Yang et al., 2021). Three of these genes encoded proteins of unknown function, while the remaining 12 genes encoded, among others, a VIVIPAROUS1 (VP1)-like transcription factor (Zm00001eb361390), a K^+^ exchanger-like protein (Zm00001eb361440), a zinc knuckle (CCHC-type) protein (Zm00001eb361470), two violaxanthin de-epoxidases (Zm00001eb361480 and Zm00001eb361490), a brassinosteroid-insensitive protein (Zm00001eb361520), a potassium channel KAT protein (Zm00001eb361550) and a phosphatidylinositol kinase (Zm00001eb361560).

**TABLE 4:** Predicted candidate genes within the *Ht3*-like region.

B73		Oh7B		M162W	
Gene ID	Predicted function	Gene ID	Predicted function	Gene ID	Predicted function
Zm00001eb361390	VP1-transcription factor	Zm00038ab369880	VP1-transcription factor	Zm00033ab382990	VP1-transcription factor
Zm00001eb361410	Myosin family protein with Dil domain	Zm00038ab369900	Myosin family protein with Dil domain	Zm00033ab383010	Myosin family protein with Dil domain
Zm00001eb361420	Mitochondrial fission protein ELM1	Zm00038ab369920	Hypothetical protein	Zm00033ab383030	NA
Zm00001eb361440	K-exchanger-like protein			Zm00033ab383040	K-exchanger-like protein
Zm00001eb361450	PLASTID MOVEMENT IMPAIRED 1-RELATED	Zm00038ab369930	PLASTID MOVEMENT IMPAIRED 1-RELATED	Zm00033ab383060	PLASTID MOVEMENT IMPAIRED 1-RELATED
Zm00001eb361460	Ras-related protein Rab7	Zm00038ab369940	Ras-related protein Rab7	Zm00033ab383070	Ras-related protein Rab7
Zm00001eb361470	zinc knuckle (CCHC-type) family protein	Zm00038ab369950	zinc knuckle (CCHC-type) family protein	Zm00033ab383080	zinc knuckle (CCHC-type) family protein
		Zm00038ab369960	uncharacterized protein	Zm00033ab383100	uncharacterized protein
Zm00001eb361480	uncharacterized protein	Zm00038ab369970	uncharacterized protein	Zm00033ab383110	uncharacterized protein
Zm00001eb361490	Violaxanthin de-epoxidase	Zm00038ab369980	Violaxanthin de-epoxidase	Zm00033ab383120	Violaxanthin de-epoxidase
Zm00001eb361500	Violaxanthin de-epoxidase			Zm00033ab383130	Violaxanthin de-epoxidase
Zm00001eb361520	Brassinosteroid insensitive	Zm00038ab369990	Brassinosteroid insensitive	Zm00033ab383140	Brassinosteroid insensitive
Zm00001eb361540	uncharacterized protein	Zm00038ab370000	uncharacterized protein	Zm00033ab383150	uncharacterized protein
Zm00001eb361550	Potassium channel KAT	Zm00038ab370010	Potassium channel KAT	Zm00033ab383160	Potassium channel KAT
Zm00001eb361560	Phosphatidylinositol kinase				
Zm00001eb361570	Hypothetical protein				

We also retrieved the corresponding regions for the mapped 577-kb *Ht3L* region from 25 sequenced founders of the NAM population (see text footnote 1). Of these 25 lines, seven were highly susceptible and another ten were highly resistant to NCLB, based on a previous report (Poland et al., 2011). The inbred lines Oh7B and M162W were the most susceptible and resistant lines, respectively (Table 4). The *Ht3L* region exhibited considerable variation in its length across inbred lines, varying from 383 kb to 590 kb (Supplementary Figure 2). Again, none of the annotated genes in these inbred lines encoded a cell wall-associated kinase. Further fine-mapping and functional testing will be required to identify the *Ht3L* causative gene conferring NCLB resistance.

## Discussion

### Rapid and reliable mapping of *Ht3L* locus

We initially genotyped the two NILs, A619*Ht3* and A619, which share ∼98.84% of their genome sequences and yet differ widely in NCLB resistance. We rapidly identified three chromosomal segments possibly associated with NCLB resistance based on the presence of SNPs between the two NILs. To narrow down the position of the *Ht3L* locus and improve NCLB resistance, we crossed the donor A619*Ht3* to the elite but highly susceptible inbred line L3162 as a recurrent parental line. The genomes of the donor A619*Ht3* and the recurrent parental line L3162 differed by sufficient SNPs to allow fine-mapping of *Ht3L*. With A619*Ht3* as the parental line, we could quickly project the three potential *Ht3L* segments from A619*Ht3/*A619 to A619*Ht3*/L3162. Given that two bulks consisted of highly resistant and susceptible individuals, respectively, we calculated the SNP-index values and looked for a region characterized by a higher index value in the resistant bulk compared to the susceptible bulk. This allowed us to detect a QTL peak on bin 8.06, which was confirmed by regional analysis using SNP-index over the three *Ht3L* candidate segments. We then attempted to continuously narrow down the *Ht3L* location by sequential fine-mapping based on recombinant-derived progeny testing (Yang et al., 2012), reaching a final interval of 577 kb.

### The deployment of multiple controls ensured the accuracy of the fine-mapping results

Stable onset of symptoms and accurate phenotypic assessment are particularly critical for QTL mapping related to NCLB resistance. Considering the uncertainty associated with natural infections, we adopted an artificial inoculation method to ensure stable and uniform environmental conditions conducive to NCLB occurrence. We therefore uniformly sprayed plants with a spore suspension, followed by spray irrigation for two days, three to four times a day, to maintain high humidity. To obtain reliable phenotypic data, we assessed DLA three times in the field four weeks after artificial inoculation, using three leaves around the ear leaf. Moreover, we planted the resistant and susceptible parental lines, along with test materials, every year as controls, which allowed us to judge the stable occurrence of NCLB. In light of the performance of the positive and negative controls in terms of resistance, we believe that our artificial inoculation method was successful and reliable.

At least four major genes and numerous QTLs have been reported for resistance to NCLB in maize (Yang et al., 2017; Zhu et al., 2021). Symptom development is influenced by both genetic factors and environmental conditions. We therefore undertook a sequential fine-mapping strategy based on recombinant-derived progeny testing (Yang et al., 2012). To minimize the influence of genetic backgrounds, we conducted five rounds of QTL fine-mapping from families derived from four to ten generations of backcrosses to ensure that each individual had an almost identical genetic background outside of the *Ht3L* region (Supplementary Figure 3). We planted all progeny derived from the same recombinant in the same plot to ensure that they experienced very similar environmental conditions. As the heterozygous and homozygous plants of the same progeny were randomly distributed in the testing plot, the difference in DLA between these two genotypes should minimally reflect any environmental influence. Generally, we backcrossed each recombinant twice to the susceptible inbred L3162 to increase the size of the mapping progeny, to further minimize both background noise and any environmental influence. In addition, we evaluated key recombinants for NCLB resistance over several years; although disease severity varied over the years, the difference in DLA between two genotypes was very stable for all key recombinants, which underscores the accuracy of the fine-mapping data presented in the current study.

A high density of molecular markers is also key to effective QTL mapping. The wide availability of genome sequences for multiple maize germplasms has driven SNP marker-based genetic mapping and QTL analysis (Chen et al., 2015; Ding et al., 2015). In the current study, we used the Maize3K and Maize6K Chips to obtain useful SNPs, from which we developed high-density KASP markers over the target *Ht3L* region. The developed markers were easy to use in each fine-mapping step and are characterized by positional accuracy, low genotyping errors, relatively low cost, and scalable flexibility in applications (Semagn et al., 2013).

### The *Ht3L* locus is independent from *Htn1*

Bin 8.05/8.06 is a hotspot for NCLB resistance, as *Ht2*, *Ht3*, *Htn1* and other QTLs against NCLB also map to this genomic interval. Recently, *Ht2* and *Ht3* were reported to be identical and allelic to *Htn1* (Yang et al., 2021). We initially anchored the *Ht3L* locus to a genomic segment of 11.23 Mb (AGPv5) in bin 8.06, flanked by the SNP markers PZE-108095959 and PZE-108110343. Notably, this 11.23-Mb segment overlapped with the known location of the *Htn1* locus. After five rounds of fine-mapping, we delineated the *Ht3L* locus to a 577-kb interval. To our surprise, the *Ht3L* locus was located ∼2.7 Mb away from *Htn1* (Figure 5). Intriguingly, when looking back over the resistance performance of different recombinants, we failed to observe any genetic contribution of the *Htn1* locus to NCLB resistance. For instance, the BC_2_F_1_ recombinant VII, which carried only the *Htn1* locus and lacked the *Ht3L* locus, did not exhibit a significant difference in DLA between its BC_4_F_1_ offspring heterozygous and homozygous for *Htn1*. Likewise, BC_2_F_1_ recombinants (No. I and II) with two loci (*Ht3L* and *Htn1*) did not display a lower DLA score than those BC_2_F_1_ recombinants (No. V) only harboring the *Ht3L* locus. The lack of phenotypic variation associated with the *Htn1* locus between A619*Ht3* and L3162 thus indicated that both lines have the same susceptible *Htn1* allele. The other possibility is that *Htn1* may lose its NCLB-resistance function due to the presence of an unidentified suppressor or the absence of a co-receptor in the L3162 background. For example, a dominant suppressor inhibiting the expression of *Ht2* was found in lines related to ‘B14’ (Ceballos and Gracen, 1989).

### The *Ht3L* locus shows extensive variation

We detected extensive genomic variation within the 577-kb *Ht3L* interval in the 25 founders of the NAM population, with a size varying from 383 kb to 590 kb. Surprisingly, no gene in this *Ht3L* region was annotated as encoding a canonical cell wall-associated kinase, in contrast to the *Ht2*/*Ht3/Htn1* locus (Yang et al., 2021). In field trials across multiple years, A619*Ht3* always exhibited highly resistance to NCLB, in sharp contrast to the highly susceptible lines A619 and L3162. Given that A619*Ht3* harbors both the resistant *Ht3L* and *Htn1* loci, we propose two possible explanations for the results of the current study: 1) the 577-kb *Ht3L* locus interval may contain a bona fide *Ht3* gene that is located ∼2.7 Mb away from the known *Ht2/Ht3/Htn1* locus; or 2) A619*Ht3* harbors a *Ht3* gene that is allelic to *Htn1*, as claimed by Yang et al. (2021), in which case the mapped *Ht3L* locus must contain a novel NCLB resistance gene. In this alternative scenario, we speculate that A619*Ht3* is unlikely to harbor another resistant allele at the reported *Ht2/Ht3* or *Htn1* loci, as only one resistance QTL was detected in our mapping populations. Since the detailed genomic sequence of the parental line A619*Ht3* is not currently available, we are still uncertain about the relationship between the currently mapped *Ht3L* locus and the reported *Ht2*/*Ht3*/*Htn1* locus. In addition, the genetic backgrounds clearly have pronounced effects on *Ht3L*-conferred NCLB resistance, as we observed wide variation in DLA scores between homozygotes and heterozygotes in the fine-mapping progeny of different parental recombinants.

### Toward the application of a novel locus in northern corn leaf blight-resistant breeding

*Exserohilum turcicum* shows clear physiological differentiation as a function of the resistance performance of inbred lines and differences of climatic conditions. Moreover, the distribution of physiological races is also complex. Twelve physiological races have been identified in China, of which races 0 and 1 are dominant (Gao et al., 2011). Because of the apparent physiological differentiation of the fungus causing NCLB, the rapid evolution of pathogen populations must be considered in NCLB-resistance breeding programs. Several resistance loci should be pyramided via marker-assisted backcrossing (MABC) to breed more resistant varieties that can combat multiple physiological races of NCLB. MABC was shown to be effective to improve the resistance of inbred lines. For example, all 63 converted lines produced through MABC by introducing nine resistant *ZmCCT* (*CONSTANS* [*CO*], *CO-like*, *TIMING OF CAB2 EXPRESSION 1* [*TOC1*]) haplotypes into seven elite maize inbred lines exhibited enhanced resistance to maize stalk rot (Li et al., 2017). The current study reveals a distinct *Ht3L* locus for NCLB resistance. Considering the substantial annual yield loss caused by NCLB, the *Ht3L* locus will be valuable in future breeding programs of NCLB-resistant maize.

## Conclusion

Mining and utilizing resistant loci/genes can greatly promote the development of resistant varieties, which will decrease yield losses and improve grain quality. With the availability of high-density SNP markers, we delimited *Ht3L* to an interval of 577kb via five rounds of sequential fine-mapping. The homozygous *Ht3L/Ht3L* genotype reduced DLA by 16.5% compared to that in lines without *Ht3L*. Our results will facilitate the cloning of the causative gene underlying the *Ht3L* locus and accelerate application of *Ht3L* in the breeding of NCLB-resistant maize varieties.

## Data availability statement

The original contributions presented in this study are included in the article/Supplementary material, further inquiries can be directed to the corresponding author/s.

## Author contributions

MJ and YW designed the experiments. JM, XL, XQ, XG, and MJ were responsible for the field tests. YG, YZ, and XL conducted genotyping. MZ and YG analyzed the data. MZ, JM, and MJ wrote the manuscript. MJ and YW supervised the project. MZ, JM, and MJ revised the manuscript in response to the reviewers. All authors read and approved the manuscript.

## Footnotes

1
http://www.maizegdb.org/
