# Supplementary material for: High-resolution mapping reveals a Ht3-like locus against northern corn leaf blight
Source: Front Plant Sci. 2022 Sep 9;13:968924. doi: 10.3389/fpls.2022.968924 (PMC9506542; doi:10.3389/fpls.2022.968924)
Supplement: Supplementary file 1 [file Data_Sheet_1.docx]

**Supplementary Table 1** Distribution of polymorphic SNPs on 10 chromosomes

|  | Chr.1 | Chr.2 | Chr.3 | Chr.4 | Chr.5 | Chr.6 | Chr.7 | Chr.8 | Chr.9 | Chr.10 | Total |
| --- | --- | --- | --- | --- | --- | --- | --- | --- | --- | --- | --- |
| Marker No. | 135 | 40 | 56 | 41 | 27 | 43 | 82 | 18 | 18 | 32 | 493 |

**Supplementary Table 2** The SNP markers in bins3.08 and 7.04 in regional association analysis

| **Chr.** | **Marker** | | **Bins** | | **Physical position**  **(bp, AGPv5)** | | **SNP-index (%)** | | **χ^2^** | ***P*-value** | |  |
| --- | --- | --- | --- | --- | --- | --- | --- | --- | --- | --- | --- | --- |
|  |  |  |  |  |  |  | **Resistance bulk** | **Susceptibility bulk** |  |  |  |  |
| 3 | | SYN32029 | | 3.08 | 217,344,023 | 41 | | 31 | 0.3 | | 0.5848 | |
|  |  | PZE-103162582 | | 3.08 | 218,460,694 | 44 | | 34 | 0.3 | | 0.5914 | |
|  |  | PZE-103163529 | | 3.08 | 219,244,567 | 48 | | 34 | 0.6 | | 0.4238 | |
|  |  | SYN33389 | | 3.08 | 220,991,974 | 48 | | 34 | 0.6 | | 0.4238 | |
|  |  | SYN19800 | | 3.08 | 222,078,614 | 48 | | 34 | 0.6 | | 0.4238 | |
|  |  | PZE-103168241 | | 3.08 | 222,387,530 | 48 | | 37 | 0.3 | | 0.5959 | |
| 7 | | SYN3222 | | 7.04 | 159,198,050 | 51 | | 65 | 1.7 | | 0.1825 | |
|  |  | SYN34204 | | 7.04 | 160,271,864 | 51 | | 62 | 1.1 | | 0.2889 | |
|  |  | PZE-107095905 | | 7.04 | 160,750,875 | 51 | | 65 | 1.7 | | 0.1825 | |
|  |  | PZE-107096020 | | 7.04 | 160,977,795 | 51 | | 65 | 1.7 | | 0.1825 | |
|  |  | PZE-107097215 | | 7.04 | 161,585,773 | 51 | | 62 | 1.1 | | 0.2889 | |
|  |  | PZE-107097708 | | 7.04 | 162,111,600 | 51 | | 62 | 1.1 | | 0.2889 | |
|  |  | PZE-107098128 | | 7.04 | 162,295,054 | 51 | | 62 | 1.1 | | 0.2889 | |
|  |  | PZE-107099124 | | 7.04 | 163,135,655 | 48 | | 55 | 0.6 | | 0.4305 | |
|  |  | PZE-107100654 | | 7.04 | 164,218,865 | 51 | | 62 | 1.1 | | 0.2889 | |
|  |  | PZE-107100880 | | 7.04 | 164,386,487 | 51 | | 62 | 1.1 | | 0.2889 | |
|  |  | PZE-107101504 | | 7.04 | 164,687,826 | 51 | | 58 | 0.6 | | 0.4283 | |
|  |  | PZE-107102089 | | 7.04 | 164,993,812 | 51 | | 62 | 1.1 | | 0.2889 | |
|  |  | PZE-107103104 | | 7.04 | 165,466,866 | 51 | | 62 | 1.1 | | 0.2889 | |

**Supplementary Table 3** Molecular markers developed in the *Ht3*-like region

| **Name** | **Location (Mb)^a^** | **Forward primer** | **Reverse primer** | **Allele**  **FAM** | **Allele**  **HEX** | **Type** |
| --- | --- | --- | --- | --- | --- | --- |
| A001802 | 143.3 | GAAGGTGACCAAGTTCATGCTCAATAGGTACAGGGCCTGTCG ^b^  GAAGGTCGGAGTCAACGGATTATCAATAGGTACAGGGCCTGTCA ^c^ | GCAGCAGAGCCAGCTAAAAGATCAA | G | A | KASP |
| A000823 | 148.1 | GAAGGTGACCAAGTTCATGCTGCTCGGAAAGCAACACCGGA ^b^  GAAGGTCGGAGTCAACGGATTGCTCGGAAAGCAACACCGGG ^c^ | GAATATGCTCGGCGTGGCTCG | A | G | KASP |
| A001155 | 149.4 | GAAGGTGACCAAGTTCATGCTGAGAAGCCAGGGATGTAATTTG ^b^  GAAGGTCGGAGTCAACGGATTCGAGAAGCCAGGGATGTAATTTA ^c^ | AGGCGGAGCAAGCTGAAC | C | T | KASP |
| A007452 | 157.7 | GAAGGTGACCAAGTTCATGCTCAACTACCGCAAGCTGCACAG ^b^  GAAGGTCGGAGTCAACGGATTGCAACTACCGCAAGCTGCACAA ^c^ | GCACGTTGCCGAAGCTGTCCTT | G | A | KASP |
| A001807 | 157.9 | GAAGGTGACCAAGTTCATGCTAGACGATTTACACCACTGTCAGG ^b^  GAAGGTCGGAGTCAACGGATTCAGACGATTTACACCACTGTCAGA ^c^ | AGAGTTTGAAGCAGCAGCCCTTGAA | G | A | KASP |
| A007453 | 158.3 | GAAGGTGACCAAGTTCATGCTCGCAAACAGTAAAAGCTCCGGTG ^b^  GAAGGTCGGAGTCAACGGATTCGCAAACAGTAAAAGCTCCGGTC ^c^ | AACGATGATGCACTTATGGGGCCAT | C | G | KASP |
| KA002076 | 158.4 | GAAGGTGACCAAGTTCATGCTGAGTGTCGTAAATGCTAGCGACT ^b^  GAAGGTCGGAGTCAACGGATTGAGTGTCGTAAATGCTAGCGACC ^c^ | ATATGCTAAAGAAGTGTCCCTGTT | T | C | KASP |
| KA002079 | 158.5 | GAAGGTGACCAAGTTCATGCTCTCTCGATGATGGTGATGTACTGG ^b^  GAAGGTCGGAGTCAACGGATTCTCTCGATGATGGTGATGTACTGA ^c^ | CTGGTTGAGGATGTTGCTTCATG | G | A | KASP |
| KA002081 | 158.7 | GAAGGTGACCAAGTTCATGCTGATATTGTGCCCTGAGTAACAAGT ^b^  GAAGGTCGGAGTCAACGGATTGATATTGTGCCCTGAGTAACAAGA ^c^ | TGGATGTAGTTCTAATGATTTCTCCTCT | T | A | KASP |
| KA002082 | 159.1 | GAAGGTGACCAAGTTCATGCTGTCAGGTGACTCTAGTTGATGCAG ^b^  GAAGGTCGGAGTCAACGGATTGTCAGGTGACTCTAGTTGATGCAA ^c^ | GCACTTGAGCTGAGGAAAATTCTT | G | A | KASP |
| KA002084 | 159.3 | GAAGGTGACCAAGTTCATGCTCTCGATCTTCATCTGCAGTGAC ^b^  GAAGGTCGGAGTCAACGGATTCTCGATCTTCATCTGCAGTGAG ^c^ | CTTCTAATGCATCTGTGGTTGCTT | C | G | KASP |
| KA002085 | 159.6 | GAAGGTGACCAAGTTCATGCTATCCTGAGGAACAAACGAGCTAA ^b^  GAAGGTCGGAGTCAACGGATTATCCTGAGGAACAAACGAGCTAG ^c^ | GTAGTTTGTGTGTGGCCTGAATC | A | G | KASP |
| KA002088 | 159.6 | GAAGGTGACCAAGTTCATGCTCCTTCAGCCAGAGTTGGTGAT ^b^  GAAGGTCGGAGTCAACGGATTCCTTCAGCCAGAGTTGGTGAC ^c^ | CTAGCTCAGTATGGCCCATGACAT | T | C | KASP |
| A007455 | 160.0 | GAAGGTGACCAAGTTCATGCTCACATCACCCTTCTCTAGTCTCA ^b^  GAAGGTCGGAGTCAACGGATTACATCACCCTTCTCTAGTCTCC ^c^ | GCGGGAGTGAGCACGCCATT | A | C | KASP |
| A007456 | 162.8 | GAAGGTGACCAAGTTCATGCTGCTACAGAAAGAAGAGAAGGCCAT ^b^  GAAGGTCGGAGTCAACGGATTCTACAGAAAGAAGAGAAGGCCAC ^c^ | GGACTCGGACCTATATATCCACCTA | A | G | KASP |
| A007457 | 163.4 | GAAGGTGACCAAGTTCATGCTGTGGACGAAGTGCTTGGAACCAA ^b^  GAAGGTCGGAGTCAACGGATTTGGACGAAGTGCTTGGAACCAG ^c^ | CCCTGGTGAACGACAGGCTGTT | T | C | KASP |
| A000827 | 165.0 | GAAGGTGACCAAGTTCATGCTGGAGAGAAGAATCTCAGCGACTCA ^b^  GAAGGTCGGAGTCAACGGATTGGAGAGAAGAATCTCAGCGACTCG ^c^ | GATGAAATGCAGCTTCTGCC | T | C | KASP |
| A001808 | 165.6 | GAAGGTGACCAAGTTCATGCTTGACGGAACACATGCACATATTTGAT ^b^  GAAGGTCGGAGTCAACGGATTGACGGAACACATGCACATATTTGAC ^c^ | TATCTCGCACCAAGCAGGGAAACAA | T | C | KASP |

1. Physical location according to B73 reference genome sequence (AGPv5).
2. Forward primer with FAM.
3. Forward primer with HEX.


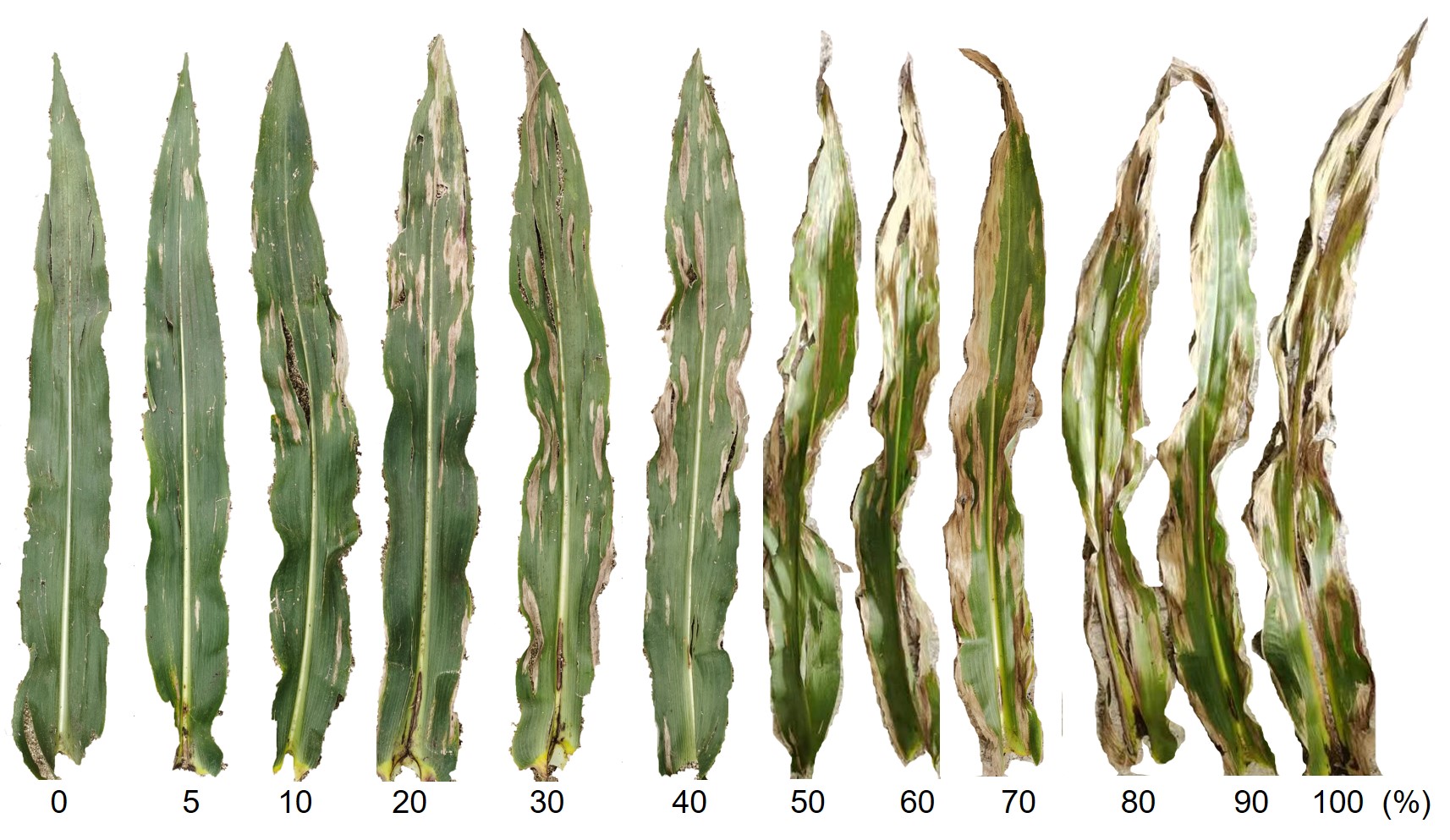


**Supplementary Figure 1**: The standards for scoring NCLB in the field


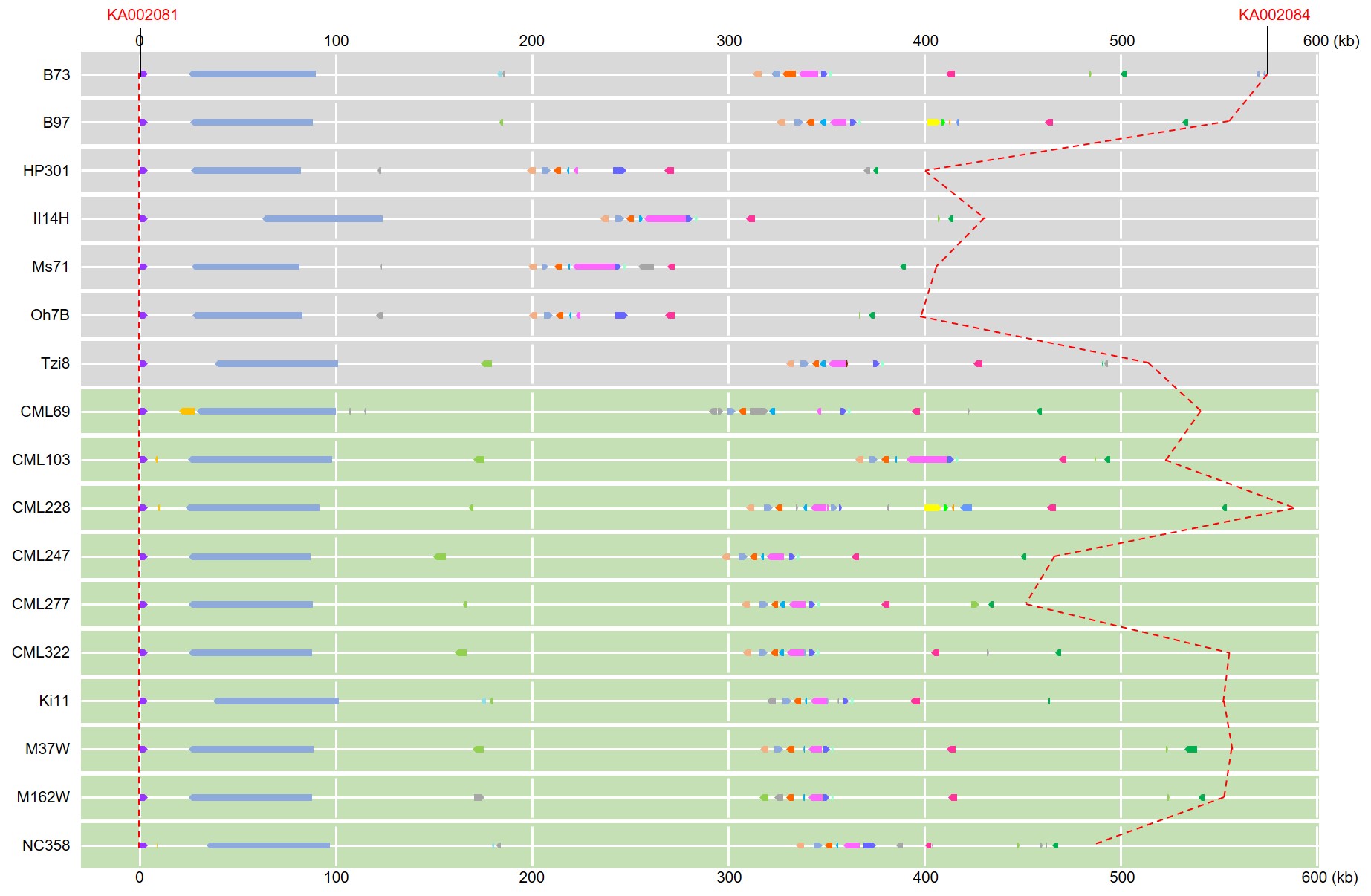


**Supplementary Figure 2** The genomic infrastructure at the mapped *Ht3*-like region.

The red dotted lines indicate the left and right boundaries of the mapped *Ht3*-like regions. From 25 NAM founders, seven NCBL susceptible and 10 NCLB resistant lines were selected and their *Ht3*-like regions are depicted as gray and green rectangles, respectively. The position and orientation of annotated genes at the *Ht3*-like region are marked as arrows with different colors.


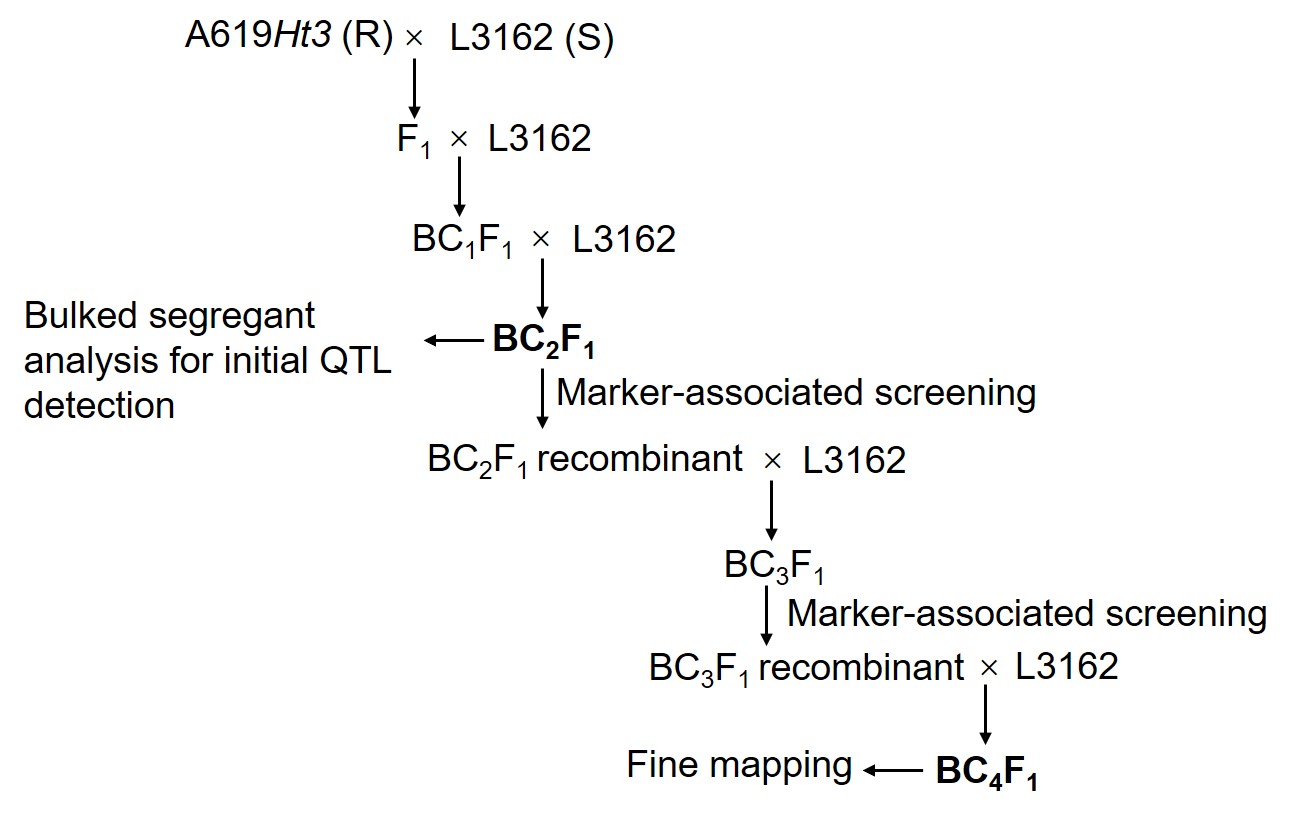


**Supplementary Figure 3** Experimental flow chart of initial QTL detection and fine mapping. The Resistant and susceptible bulks were composed of 29 highly resistant and 29 highly susceptible individuals from the BC_2_F_1_ population, respectively. Two bulks were individually evaluated for NCLB resistance and genotyped with Maize6K chip for initial QTL identification. Fine mapping of the *Ht3* locus was initiated from the BC_4_F_1_ generation and ended at the BC_10_F_1_ generation.
